# Maternal, Fetal and Neonatal Outcomes Related to Recreational Cannabis Use during Pregnancy: Analysis of a Real-World Clinical Data Warehouse between 2010 and 2019

**DOI:** 10.3390/ijerph20176686

**Published:** 2023-08-30

**Authors:** Emilie Bouquet, Pascal Blouin, Marie-Christine Pérault-Pochat, Caroline Carlier-Guérin, Frédéric Millot, Jean-Baptiste Ricco, Joe De Keizer, Stéphanie Pain, Farid Guétarni

**Affiliations:** 1 Addictovigilance Center, Department of Clinical Pharmacology, Poitiers University Hospital, 86000 Poitiers, France; 2 French National Institute of Health and Medical Research (INSERM) U1084, Experimental and Clinical Neurosciences Laboratory, University of Poitiers, CEDEX 9, 86073 Poitiers, France; 3 Clinical Investigation Center CIC1402, INSERM, Poitiers University Hospital, University of Poitiers, 86000 Poitiers, France; 4 Department of Clinical Research and Innovation, Poitiers University Hospital, 86000 Poitiers, France; 5 Department of Obstetrics and Gynecology, Poitiers University Hospital, 86000 Poitiers, France; 6 Department of Pediatrics, Poitiers University Hospital, 86000 Poitiers, France

**Keywords:** recreational cannabis, adverse events, pregnancy, perinatal

## Abstract

Background: Cannabis is the main illicit psychoactive substance used in French childbearing women and very few data are available about adverse events (AEs) related to its use during pregnancy. The aim of this study was to evaluate the association between recreational cannabis use during pregnancy and adverse outcomes from a real-world clinical data warehouse. Methods: Data from the Poitiers University Hospital warehouse were analyzed between 1 January 2010 and 31 December 2019. Logistic regression models were used to evaluate associations between outcomes in three prenatal user groups: cannabis alone ± tobacco (C ± T) (*n* = 123), tobacco alone (T) (*n* = 191) and controls (CTRL) (*n* = 355). Results: Pregnant women in the C ± T group were younger (mean age: 25.5 ± 5.7 years), had lower pre-pregnancy body mass index (22.8 ± 5.5 kg/m^2^), more psychiatric history (17.5%) and were more likely to benefit from universal free health-care coverage (18.2%) than those in the T and CTRL groups. Cannabis use increases the occurrence of voluntary interruption of pregnancy, at least one AE during pregnancy, at least one neonatal AE, the composite adverse pregnancy outcome over 28, prematurity and small for gestational age. Conclusion: Given the trivialization of recreational cannabis use during pregnancy, there is an urgent need to communicate on AEs of cannabis use during pregnancy.

## 1. Introduction

Epidemiological data have shown a trivialization of recreational cannabis use worldwide, notably during pregnancy. In France, cannabis is the main illicit psychoactive substance used in childbearing women. In 2017, 4% of women aged 18 to 34 years reported a regular cannabis use, that is, at least ten times a month [1]. According to the latest national perinatal survey from 2021, 1.1% of women interviewed in postpartum care reported cannabis use during pregnancy, of whom 41% reported using it more than six times a month, but this prevalence is probably underestimated due to under-reporting [2]. In addition, the French pharmaco-epidemiological survey OPPIDUM (Observation of Illegal Drugs and Misuse of Psychotropic Medications) showed a significant increase in the use of cannabis from 23.5% to 37.1% between 2005 and 2018 in pregnant women managed in specialized care centers dedicated to drug dependence [3]. However, these surveys did not provide data on maternal, fetal or neonatal complications related to recreational cannabis use during pregnancy, and very few data are available in France. The recent analysis of notifications from the French Addictovigilance network over the past ten years has shown that the main events reported among pregnant cannabis users were psychiatric (*n* = 96, 64.9%), mainly use disorders (*n* = 89, 60.1%), dependence (*n* = 54, 36.5%) and abuse (*n* = 21, 14.2%) [4]. The main fetal events were heart rhythm disorders (*n* = 25, 16.9%) and intrauterine growth retardation (*n* = 20, 13.5%) and the main neonatal events were growth retardations (*n* = 39, 26.3%) and prematurity (*n* = 32, 21.6%) [4]. Twelve cases of congenital malformations were described and four intrauterine or neonatal deaths [4]. However, one of the limitations of this study is the under-reporting of adverse events (AEs).

Cannabis is a plant containing over 120 phytocannabinoids, the main being tetrahydrocannabinol (THC). It is involved in the regulation of the hypothalamic-pituitary-gonadal axis, as it reduces the release of gonadotrophin-releasing hormone (GnRH), luteinizing hormone (LH) and possibly follicle-stimulating hormone (FSH), which are necessary for follicular growth, ovulation, maintenance of corpus luteum and for the synthesis of progesterone, essential for the maintenance of early pregnancy [5]. Furthermore, THC increases mRNA expression of aromatase, a key enzyme involved in estrogen biosynthesis and necessary for placentation, via the estrogen receptor alpha-mediated signaling [6]. This, in turn, reduces the plasmatic levels of estradiol, testosterone and progesterone [5]. Because of its lipophilicity, THC can cross the placenta to enter the fetal bloodstream [7]. THC has been shown to significantly increase N-arachidonoylphosphatidylethanolamine-specific phospholipase D (NAPE-PLD) activity and to inhibit fatty acid amine hydrolase (FAAH), resulting in higher concentrations of endocannabinoids within the placenta; placental changes such as labyrinth zone expansion, reduced fetal capillary area, trophoblast and decidual dysfunction; phenotypic changes in trophoblasts; and reduced glucose transporter-1 expression and glucocorticoid receptors, contributing to intrauterine growth restriction [7]. Consequently, prenatal exposure to cannabis may deregulate the endocannabinoid system, which plays a key role in pregnancy. Furthermore, the mean THC levels have considerably risen these last ten years in France as well as worldwide [8,9], which may increase the risk of AEs.

The aim of this study was to evaluate the association between recreational cannabis use during pregnancy and maternal, fetal and neonatal outcomes from a real-world Hospital Clinical Data warehouse.

## 2. Materials and Methods

In this retrospective observational study, data from pregnant women and their children followed in the level III maternity of Poitiers University Hospital were analyzed between 1 January 2010 and 31 December 2019. All data were abstracted from this database.

### 2.1. Data Source

This study was established using the electronic clinical data warehouse of Poitiers University Hospital “eHOP” (Entrepôt des données de l’Hôpital) [10]. “eHOP” is a real-world clinical health Big Data warehouse routinely including both heterogeneous clinical data, such as hospitalization and consultation records; results of medical imagery; biological analysis, in particular toxicological tests; drug prescriptions; administrative, data such as hospital discharge diagnoses coded according to the International Classification of Diseases, 10th Revision (ICD-10); medical procedures coded according to the French medical classification for clinical procedures (CCAM), a unique identifier linking records of mothers and children; and data of reimbursement (health insurance, complementary universal health insurance CMU-c, state medical assistance AME).

### 2.2. Study Population and Exposures

**Figure 1:**
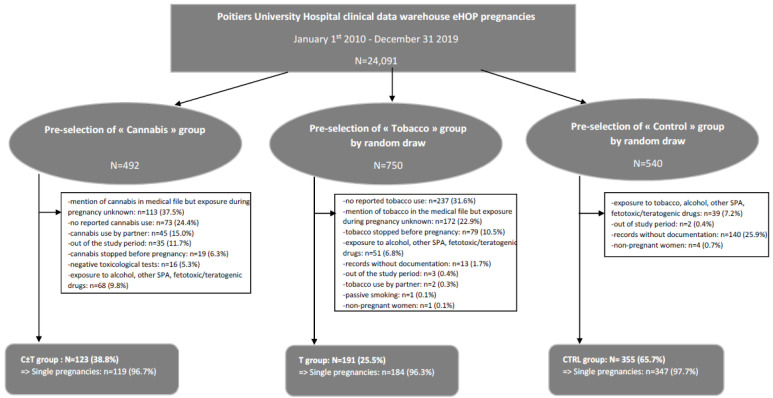
Flowchart of the study. Abbreviations: PW: pregnant women, C: cannabis, T: tobacco, CTRL: control, SPA: psychoactive substances.

Three subgroups of pregnant women and their children admitted in the level III maternity of Poitiers University Hospital between 1 January 2010 and 31 December 2019 were preselected according to the following criteria (Figure 1):

- The subgroup “Cannabis” refers to those who were prenatally exposed to recreational cannabis, using diagnostic ICD-10 codes and the medical procedure CCAM corresponding to care of pregnant women and deliveries and either textual research in the records for different terms identifying cannabis exposure (cannabis, marijuana, etc.), results of toxicological tests revealing cannabis exposure or ICD-10 codes for hospital discharge diagnoses related to cannabis use.
- The subgroup “Tobacco” refers to those who were prenatally exposed to tobacco, using diagnostic ICD-10 codes and the medical procedure CCAM corresponding to care of pregnant women and deliveries and either textual research in the records for different terms identifying tobacco exposure (tobacco, cigarettes, etc.) or ICD-10 codes for hospital discharge diagnoses related to tobacco use.
- The subgroup “Control” refers to those who were not prenatally exposed to cannabis, tobacco, alcohol or any other medical or non-medical psychoactive substance, using diagnostic ICD-10 codes and the medical procedure CCAM corresponding to care of pregnant women and deliveries and excluding terms and ICD-10 codes corresponding to an exposure to cannabis, tobacco, alcohol or any other medical or non-medical psychoactive substance.

Records of mothers and their children were linked through a unique identifier.

As cannabis is frequently associated with tobacco, in particular when inhaling joints, three groups were then constituted in order to estimate the effects of cannabis independently of tobacco:

- The C ± T group, including pregnant women and their children exposed to cannabis alone or in association with tobacco.
- The T group, including pregnant women and their children exposed to tobacco alone.
- The CTRL group, including pregnant women and their children not exposed to either cannabis or tobacco.

After selecting all the cases prenatally exposed to cannabis alone or in association with tobacco (group C ± T), we selected cases prenatally exposed to tobacco alone (group T) and the group control (CTRL) using a random draw, carrying out a sampling test against controls with a power of 0.9 that was two-sided and had a ratio of 1:5 to increase statistical power.

Exclusion criteria were:

- No pregnancy, pregnancy out of the study period or records without documentation.
- Exposure to psychoactive substances other than cannabis and tobacco such as alcohol, cocaine, etc., or exposure to fetotoxic, teratogenic or other drugs increasing the risk of maternal, fetal or neonatal complications.
- Specifically for the C ± T group: no reported cannabis use, cannabis stopped before pregnancy, mention of cannabis in the medical file but exposure during pregnancy unknown, cannabis use by partner only, no reported use or negative toxicological tests (notably, in the absence of reported use).
- Specifically for the T group: no reported tobacco use, tobacco stopped before pregnancy, mention of tobacco in the medical file but exposure during pregnancy unknown, tobacco use by the partner only or passive smoking.
- Specifically for the CTRL group: exposure to any psychoactive substances (cannabis, tobacco, alcohol, cocaine, etc.) or to fetotoxic, teratogenic or other drugs increasing the risk of maternal, fetal or neonatal complications.

Selected records were reviewed in order to exclude wrongly selected files due to coding errors or errors in hospitalization reports.

Exposures were grouped into three categories: during the first, the second and the third trimester of pregnancy. When consumption was reported throughout the pregnancy, it was counted for each trimester. Tobacco exposure daily and ≥10 cigarettes/day were defined at each trimester of pregnancy according to categories usually used by the French Monitoring Center for Drugs and Drug Addiction (Observatoire français des drogues et des toxicomanies) and the French National Perinatal Survey 2010 [11,12]. Cannabis exposure daily and ≥10 times/day were defined by analogy with tobacco use.

For the analysis of adverse outcomes, only single pregnancies were considered because twin pregnancies are associated with an increased risk.

### 2.3. Outcomes

The outcomes were events occurring during pregnancy, delivery and immediate postpartum period: voluntary interruption of pregnancy, at least one AE during pregnancy when women experienced one or more AEs during pregnancy, composite adverse pregnancy outcome defined by Rodriguez et al. [13], gestational diabetes, at least one neonatal AE when newborns experienced one or more AEs after delivery, prematurity, small for gestational age, congenital malformations and other severe adverse outcomes.

The composite adverse pregnancy outcome included the presence of at least one of the following events: spontaneous preterm birth (<37 weeks of gestation) with or without intact membranes, gestational hypertension, pre-eclampsia, eclampsia, HELLP syndrome, stillbirth or small for gestational age. It has already been used to evaluate adverse pregnancy outcomes in young pregnant women who used cannabis [13].

Prematurity was defined as gestational age less than 37 weeks of gestation.

Small for gestational age was defined as the tenth percentile of birthweight by week of gestation and sex according to French growth curves (https://www.audipog.net/, accessed on 18 December 2020).

### 2.4. Covariates

Potential confounders considered in this study included maternal age at birth, pre-pregnancy body mass index (BMI), gyneco-obstetrical history, psychiatric history, nulliparity, health insurance (no mutual insurance, mutual insurance, complementary universal health coverage (CMUc) or state medical aid (AME)) and sex of the newborn.

### 2.5. Data Analysis

The first stage of analysis was to describe socio-demographic and health characteristics of the study population as well as those of the exposures. Quantitative variables were presented as mean (standard deviation, range), while qualitative variables were presented as numbers (%).

The characteristics of the three prenatal user groups C ± T, T and CTRL were compared using Chi-square or Fisher tests for the comparison of qualitative variables and Student or Mann–Whitney tests for the comparison of quantitative variables.

Logistic regression models were used to evaluate associations between outcomes in the groups C ± T, T and CTRL.

As women in the C ± T group were younger and cannabis use is more common among young people [12], an interaction was systematically evaluated between maternal age at birth and exposures. When an interaction was found and in order to distribute the groups fairly uniformly, maternal age at birth was dichotomized in two classes: “<25 years and ≥25 years” for the analysis of voluntary interruption of pregnancy and “≤28 years and >28 years” for the analysis of the composite adverse pregnancy outcome and prematurity.

Because obesity, defined by a BMI ≥ 30 kg/m^2^, is a known risk factor for gestational diabetes, fetal growth and congenital malformations [14,15], the unadjusted and adjusted logistic regression models for gestational diabetes, at least one neonatal AE, small for gestational age and congenital malformations were performed on a sub-population with complete pre-pregnancy BMI data (because of their frequent missing values).

Sensitivity analyses were performed after imputation for missing data with the R package Multiple Imputation by Chained Equation (MICE) to evaluate the robustness of the associations.

Analyses were performed using the software R (version 4.2.0, R core Team 2022).

### 2.6. Ethical Statement

The Poitiers University Hospital “eHOP” warehouse is registered in the French Data Protection Agency Commission Nationale Informatique et Liberté (CNIL)/General Data Protection Regulation (RGPD) (Authorization CHU86-R2018-09-01). All patients were informed via a welcome booklet upon their admission at the hospital and could oppose to the use of their data for research. This research was authorized by the CNIL (Authorization CHU86-R2019-12-01, 13 December 2019).

## 3. Results

### 3.1. Study Population

A search of the clinical data warehouse of Poitiers University Hospital over the study period enabled us to preselect 1782 records, of whom 669 (37.5%) were included: 123 in the C ± T group, 191 in the T group and 355 in the CTRL group. The records of the C ± T and T groups excluded were those for which cannabis and/or tobacco was used outside pregnancy and/or stopped before pregnancy and those with other concomitant psychoactive substances, such as alcohol, cocaine, etc., for which it was specified that women did not use cannabis and or tobacco or in which it was indicated that it was only her partner who did. The reports of the CTRL group excluded were those for which any psychoactive substances were consumed. All records without documentation were also excluded (Figure 1).

Women in the C ± T group were younger (mean age: 25.5 ± 5.7 years); had lower pre-pregnancy body mass index (22.8 ± 5.5 kg/m^2^); and more psychiatric (14.6%), abuse/dependence (83.7%) and gyneco-obstetrical (48.8%) history. They were more likely to be nulliparous (50.4%) and to have a lower socio-economic status (universal free health-care coverage: 16.3%, no mutual insurance: 52.8%) than those in the T and CTRL groups (Table 1).

### 3.2. Exposure

Among women in the C ± T group for whom data are available, daily use of cannabis concerned 31 (25.2%) of them in the first trimester of pregnancy, including 2 (1.6%) who engaged in use more than ten times a day; 17 (28.8%) in the second trimester; and 18 (36.0%) in the third trimester (Table 2). The reported daily tobacco smoking was less frequent than in the C ± T group in the first and the second trimester of pregnancy (*n* = 53, 49.5% versus *n* = 128, 67.0%, and *n* = 28, 45.2% versus *n* = 91, 63.2%, respectively) (Table 2). Nevertheless, no difference was found for high consumption (≥10 cigarettes/day) between the two groups. When this data was available, cannabis was inhaled in all cases (data not shown). In very few cases (1.6%), toxicological tests were performed and confirmed cannabis use in the C ± T group.

### 3.3. Outcomes

**Figure 2:**
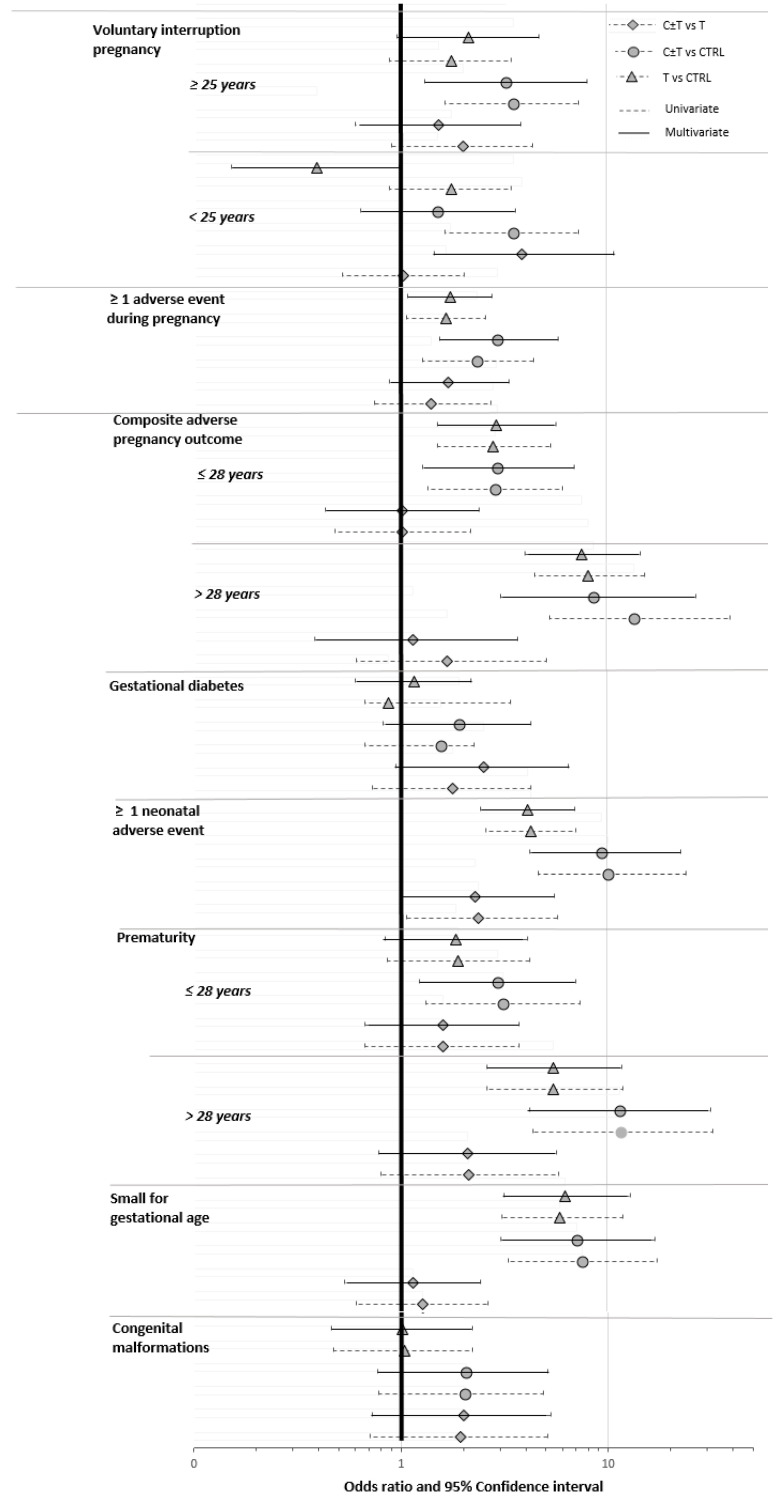
Univariate and multivariate odds ratios and 95% confidence intervals. All multivariate models adjusted on confounding factors. Confounding factors considered were maternal age at birth, pre-pregnancy body mass index, gyneco-obstetrical history, psychiatric history, nulliparity, health insurance and sex of the newborn.

The results of logistic regression analysis are presented in Figure 2 and Supplementary Tables S1–S8.

#### 3.3.1. Voluntary Interruption of Pregnancy

The prevalence of voluntary interruption of pregnancy was higher in the C ± T than in the T and CTRL groups (32.5% vs. 14.7% and 14.9%). Among women under 25, an increased risk of voluntary interruption of pregnancy was observed in the C ± T group compared to the T group (adjusted odds ratio (aOR), 3.86, 95% confidence interval (CI), 1.46–10.87) and a decreased risk in the T group compared to the CTRL group (aOR, 0.39, 95% CI, 0.15–0.98). Over 25, an increased risk of voluntary interruption of pregnancy was shown in the C ± T group compared to the CTRL group (aOR, 3.22, 95% CI, 1.30–8.02, not significant compared to the T group) (Figure 2, Supplementary Table S1).

#### 3.3.2. At Least One AE during Pregnancy

The prevalence of at least one AE during pregnancy was higher in the C ± T and T groups than in the CTRL group (56.9% and 58.1% vs. 35.8%). Women in the C ± T group had higher odds for occurrence of at least one AE during pregnancy than those in the T group compared to the CTRL group (OR, 2.34, 95% CI, 1.28–4.43, aOR, 2.93, 95% CI, 1.54–5.79 and OR, 1.66, 95% CI, 1.07–2.59, aOR, 1.73, 95% CI, 1.08–2.78, respectively) (Figure 2, Supplementary Table S2).

#### 3.3.3. Composite Adverse Pregnancy Outcome

The composite adverse pregnancy outcome was more common in the C ± T and the T groups than in the CTRL group (50.4% and 44.0% vs. 11.3%). After adjustment on confounding factors, the composite adverse pregnancy outcome remained similar between the C ± T and the T groups among women under 28 (aOR = 2.94, 95% CI= 1.27–6.94 and aOR = 2.90, 95% CI = 1.51–5.70, respectively). Among women over 28, the composite adverse pregnancy outcome remained higher in the C ± T group than in the T group compared to the CTRL group (aOR, 8.64, 95% CI, 3.04–27.11 and aOR, 7.56, 95% CI, 4.03–14.56 respectively) (Figure 2, Supplementary Table S3).

#### 3.3.4. Gestational Diabetes

Gestational diabetes was less common among women in the C ± T group than in the T and CTRL groups (8.9% vs. 9.9% and 9.3%). Women in C ± T had no significant increased risk of gestational diabetes compared to the T and the CTRL groups, and those in the T group had no significant increased risk of gestational diabetes compared to those in the CTRL group (Figure 2, Supplementary Table S4).

#### 3.3.5. At Least One Neonatal AE

The prevalence of at least one neonatal AE was higher in the C ± T and T groups than in the CTRL group (25.2% and 34.0% vs. 13.0%). Children of mothers in the group C ± T had an increased risk of at least one neonatal AE compared to those in the T and CTRL groups (OR, 2.38, 95% CI, 1.07–5.76, aOR, 2.29, 95% CI, 1.02–5.58 and OR, 10.11, 95% CI, 4.65–24.04, aOR, 9.39, 95% CI, 4.22–22.81, respectively). Those in the group T also had an increased risk of at least one neonatal AE but it was lower (OR, 4.24, 95% CI, 2.57–7.08, aOR, 4.10, 95% CI, 2.43–7.00) compared to the CTRL group (Figure 2, Supplementary Table S5).

#### 3.3.6. Prematurity

The prevalence of prematurity was higher in the C ± T and T groups than in the CTRL group (20.3% and 19.9% vs. 7.9%). Among women under 28, an increased risk of prematurity was observed in the group C ± T compared to the CTRL group (OR, 3.11, 95% CI, 1.32–7.41, aOR, 2.93, 95% CI, 1.23–7.03), while no significant association was found in the T group after adjustment on confounding factors. Among women over 28, those in the C ± T group presented higher odds of prematurity that those in the T-group when compared to the CTRL group (OR, 11.7, 95% CI, 4.36–32.65, aOR, 11.47, 95% CI, 4.23–31.87 and OR, 5.50, 95% CI, 2.62–11.90, aOR = 5.47, 95% CI, 2.60–11.84) (Figure 2, Supplementary Table S6).

#### 3.3.7. Small for Gestational Age

The prevalence of small for gestational age was higher in the C ± T and T groups than in the CTRL group (17.9% and 24.6% vs. 5.6%). Children of mothers in the group C ± T had higher odds for small for gestational age than those in the group T compared to those in the CTRL group (respectively, OR, 7.56, 95% CI, 3.32–17.55, aOR, 7.15, 95% CI, 3.04–17.16 and OR, 5.91, 95% CI, 3.09–11.87, aOR, 6.26, 95% CI, 3.17–13.03) (Figure 2, Supplementary Table S7).

#### 3.3.8. Congenital Malformations

The prevalence of congenital malformations was higher in the C ± T and T groups than in the CTRL group (6.5% and 6.3% vs. 3.6%). No significant increased risk of congenital malformations was shown in the C ± T, T and CTRL groups (Figure 2, Supplementary Table S8), but the effect size was small (*n* = 10 in the C ± T group versus *n* = 13 in the T-group and N = 24 in the CTRL group). In the C ± T group, it was not possible to define a specific pattern of malformations (Supplementary Table S9), and in two cases, they led to a medical termination of pregnancy.

#### 3.3.9. Other Severe Adverse Outcomes

Other severe adverse outcomes were also found in the C ± T group, such as threat of preterm delivery (*n* = 11), cannabis dependence/withdrawal syndrome (*n* = 10), placental abnormalities (*n* = 7), oligoamnios/anamnios (*n* = 7), hypertension/eclampsia/pre-eclampsia/HELLP syndrome (*n* = 5), maternal haemorrhagic stroke (*n* = 1), maternal reversible posterior encephalopathy syndrome (*n* = 1), fetal heart rhythm disorders (*n* = 12), Apgar < 7 at 1 min (*n* = 6), respiratory failure (*n* = 4), hypoglycaemia (*n* = 3), bradycardia (*n* = 2), stillbirth (*n* = 3) and in utero fetal death (*n* = 1) (Supplementary Table S10).

#### 3.3.10. Sensitivity Analysis

Sensitivity analysis after imputation of missing data showed similar results for all outcomes except for small for gestational age. For this outcome, the effect of cannabis and tobacco disappeared after imputation of missing data. Children of mothers in the group C ± T had a very low increased risk for small for gestational age (aOR, 1.05, 95% CI, 1.00–1.10), whereas those of mothers in the T group had no increased risk (aOR, 0.97, 95% CI, 0.93–1.01). This may be explained by the high number of missing values for pre-pregnancy BMI, which led us to consider the sub-population with complete pre-pregnancy BMI data.

## 4. Discussion

The adverse effects of tobacco smoking during pregnancy are well known, which is not the case of those of cannabis [16]. This study shows the harmful effects of cannabis use during pregnancy. It increases the risk of voluntary interruption of pregnancy, at least one AE during pregnancy, the composite adverse pregnancy outcome in women over 28, at least one neonatal AE, prematurity and small for gestational age.

Women in the C ± T group were younger than those in groups T and CTRL, which is in agreement with the literature [12,17,18] and was taken into account in multivariate analyses.

The increased risk of voluntary interruption of pregnancy was expected in the C ± T group. A previous French study found a higher risk of unplanned pregnancies (aOR, 1.61, 95% CI, 1.00–2.58) and abortion (aOR, 1.77, 95% CI, 1.26–2.49) in women who have used cannabis in their lifetime compared to non-users [19].

In addition, pregnant women in the C ± T group were more likely to experience at least one AE than those in the T group. The occurrence of composite adverse pregnancy outcomes was similar in the C ± T and the T groups compared to the CTRL group among women under 28 years of age, which is consistent with the results of Rodriguez et al., which evaluated the same composite outcome in youngest pregnant women with self-reported cannabis use (13–22 years old) [13]. In our study, we also assessed this composite adverse pregnancy outcome in older women (over 28) and have shown a higher odds ratio in the C ± T group than in the T group when compared to the CTRL group (aOR = 8.64, 95% CI = 3.04–27.11 and aOR, 7.56, 95% CI = 4.03–14.56, respectively). Thus, after controlling for confounding factors, older women in the C ± T group have a higher risk for this composite adverse pregnancy outcome compared to the T group.

We observed that the odds ratio of prematurity for women in the C ± T group compared to the CTRL group was twice as high as for women in the T group compared to the CTRL group over the age of 28. Studies that have assessed the risk of prematurity in pregnant women who use cannabis have shown controversial results [20]. Our results are consistent with the most recent studies covering the same time period that have shown an increased risk [21,22,23].

We found no effect of cannabis use during pregnancy on the risk of gestational diabetes. In the literature, the risk of gestational diabetes in pregnant women who use cannabis was not significant or very moderately decreased [20].

Our results also showed that cannabis use during pregnancy has harmful effects on infants. The children of mothers in the group C ± T were more than twice as likely to live through at least one AE compared to those in the T group. They also had an increased risk of small for gestational age, which has already been described [12,23,24] and may be explained by an impairment of the placentation and angiogenesis, an increase in placental vascular resistance [7,25]. THC has also been shown to significantly decrease glucocorticoids and transporters GLUT1, which suggests that it may reduce the disposability of glucose, the main energy substrate for the fetus [7]. Furthermore, a dose-response between cannabis use during pregnancy and birth weight has already been suggested [24].

These adverse maternal, fetal and neonatal outcomes can also be explained by the hormonal modifications induced by cannabis. Estrogen has been shown to be involved in the utero-placental vascular morphogenesis, in particular uterine angiogenesis, spiral artery remodeling, uterine vasodilatation and hemodynamic adaptation throughout pregnancy [26]. Progesterone is involved in decidualization and differentiation of endometrial stromal cells. Both regulate the activity of NK cells in the uterus [27]. Thus, perturbations of their signaling can lead to several pregnancy complications such as miscarriage, pre-eclampsia and preterm birth. In addition, gestation duration has been inversely associated with estradiol, oestriol and progesterone maternal plasmatic levels, whereas birth weight has been positively associated [28].

No increased risk of congenital malformations was observed in both the C ± T and T groups compared to controls. This may be explained by the small sample size and the recruitment at the maternity of the Poitiers University Hospital, which is a reference center that coordinates the region’s prenatal diagnosis activity. Nevertheless, chronic cannabis use has been shown to be associated with lower serum folic acid concentrations compared with lower levels of use or no use at all [29]. Folic acid has been shown to reduce the risk of some congenital malformations and may play a fundamental role in the regulation of trophoblast invasion and placental development in early pregnancy [30].

Women in the T group smoked tobacco slightly more than those in the C ± T group. Nevertheless, this result should be taken with caution since in our study cannabis was in all cases inhaled. We cannot rule out an undeclared tobacco use in association with cannabis.

Our study, based on a real-life database, allows limited under-reporting of AEs. The textual research with different terms corresponding to cannabis use makes it possible to identify almost all records with cannabis exposure reported in the medical file of pregnant women. Nevertheless, we cannot exclude some under-reporting of cannabis use by pregnant women to practitioners. Some pregnant women may not declare their consumption because they fear stigma and involvement of child protective services [31]. Therefore, other pregnant women are likely to have been exposed to cannabis. Few toxicological tests were carried out because it is not allowed in current practice to systematically request them from all pregnant women. Unfortunately, some data were likely to be incomplete because they were not recorded in the medical file and it was not possible to retrieve them retrospectively, as for example the pre-pregnancy BMI and early spontaneous miscarriages. A significant number of records were excluded in the absence of confirmed exposure to cannabis during pregnancy. Thus, the sample size was small and did not allow for the analysis of some outcomes by logistic regression, such as maternal hypertension and eclampsia/pre-eclampsia/HELLP syndrome, anamnios/oligoanamnios, placental abnormalities, Apgar < 7 at 1 min or neonatal respiratory failure, or to assess them by cannabis use characteristics such as frequency of use. Another recently published retrospective study using California linked hospital discharge–vital statistics data showed a significant association between exposure to cannabis during pregnancy and the risk of gestational hypertension (aOR, 1.19, 95% CI, 1.06–1.34), pre-eclampsia (aOR, 1.16, 95% CI, 1.06–1.34), neonatal respiratory distress (aOR, 1.16, 95% CI, 1.07–1.27) and neonatal intensive care unit admission (aOR, 1.24, 95% CI, 1.16–1.33) [23].

This study was conducted in the level III maternity of Poitiers University Hospital, specialized in the management of high-risk pregnancies. Therefore, these results cannot be extrapolated to all pregnancies exposed to cannabis.

In many published studies that have evaluated adverse outcomes related to cannabis use during pregnancy, concomitant consumption is not always specified. However, data from the French Addictovigilance network have highlighted frequent polyconsumption among pregnant women who use cannabis [3,4]. We have chosen to exclude pregnancies that were also exposed to other psychoactive, fetotoxic or teratogenic substances and other drugs known to have deleterious effects on pregnancy for the analysis of adverse outcomes, except tobacco frequently associated with cannabis, in order to limit the risk of bias related to their use.

This study also described other severe events, such as threat of preterm delivery, placental abnormalities, oligoamnios/anamnios, hypertension/eclampsia/pre-eclampsia, HELLP syndrome, fetal heart rhythm disorders, Apgar < 7 at 1 min, stillbirth, utero fetal death and dependence, withdrawal syndrome, hemorrhagic stroke, reversible posterior encephalopathy syndrome, respiratory failure and bradycardia, that have been previously described among cannabis users [32].

## 5. Conclusions

In this real-world study, cannabis use during pregnancy increases the occurrence of voluntary interruption of pregnancy, at least one AE during pregnancy, composite adverse pregnancy outcome among older women, at least one neonatal AE, prematurity and small for gestational age. These AEs should lead practitioners to systematically question all pregnant women about cannabis use and monitor them closely. Given the trivialization of recreational cannabis consumption during pregnancy, it is urgent to communicate about the risk of cannabis during pregnancy and to inform the public, in particular the future mothers.

## Figures and Tables

**Table 1:** Maternal and pregnancy characteristics by groups.

	Group			*p*
	C ± T (*N* = 123)	T (*N* = 191)	CTRL (*N* = 355)	
Maternal age at birth (years)	*N* = 123	*N* = 191	*N* = 354	**<0.0001**
	Mean: 25.5	Mean: 28.4	Mean: 29.5	
	SD: 5.7	SD: 5.8	SD: 6.0	
	Range: 14–40	Range: 18–44	Range: 15–45	
Pre-pregnancy BMI (kg/m^2^)	*N* = 70	*N* = 151	*N* = 206	**0.004**
	Mean: 22.8	Mean: 25.9	Mean: 24.7	
	SD: 5.5	SD: 7.4	SD: 6.0	
	Range: 16–39	Range: 15–60	Range: 16–54	
Pre-pregnancy BMI (kg/m^2^)	*N* = 70	*N* = 151	*N* = 354	**0.05**
<30 kg/m^2^	59 (84.3%)	113 (74.8%)	174 (84.5%)	
≥30 kg/m^2^	11 (15.7%)	38 (25.2%)	32 (15.5%)	
Social protection	*N* = 110	*N* = 185	*N* = 344	**<0.0001**
No mutual insurance	65 (52.8%)	69 (36.1%)	115 (32.4%)	
Mutual insurance	25 (20.3%)	98 (51.3%)	201 (56.7%)	
CMUc/AME	20 (16.3%)	18 (9.4%)	28 (8.0%)	
Psychiatric history	*N* = 103	*N* = 159	*N* = 251	**<0.0001**
yes	18 (14.6%)	6 (3.1%)	4 (1.1%)	
no	85 (69.1%)	153 (80.1%)	247 (69.6%)	
Abuse/dependence history	*N* = 113	*N* = 172	*N* = 353	**<0.0001**
yes	103 (83.7%)	73 (38.2%)	3 (0.8%)	
no	10 (8.1%)	99 (51.8%)	350 (98.6%)	
Gyneco-obstetrical history	*N* = 62	*N* = 191	*N* = 309	**<0.001**
yes	60 (48.8%)	81 (42.4%)	105 (29.6%)	
no	62 (43.1%)	110 (57.6%)	204 (57.5%)	
Nulliparous	*N* = 105	*N* = 181	*N* = 301	**0.0004**
yes	62 (50.4%)	87 (45.5%)	129 (36.3%)	
no	43 (35.0%)	94 (49.2%)	172 (48.5%)	

Quantitative variables reported as mean, standard deviation (SD) and range. Qualitative variables reported as number (*N*) and percentage (%). Abbreviation: BMI: body-mass index (kg/m^2^), C: cannabis, T: tobacco, CTRL: control. *p*-value: Student or Mann–Whitney tests for comparison of quantitative variables, Chi-square or Fisher tests for comparison of qualitative variables. *p*-value in bold: significant effect.

**Table 2:** Characteristics of exposures during pregnancy.

Exposures	C ± T Group	T Group	*p*
**Cannabis use during pregnancy**			
**In the first trimester**	***N* = 123**		
yes	123 (100%)		
no	0 (0%)		
daily	31 (25.2%)		
≥10 times/day	2 (1.6%)		
**In the second trimester**	*N* = 76		
yes	59 (77.6%)		
no	9 (11.8%)		
unknown	8 (10.6)		
daily	17 (28.8%)		
≥10 times/day	0 (0.0%)		
**In the third trimester**	*N* = 73		
yes	50 (68.5%)		
no	15 (20.5%)		
unknown	8 (11.0%)		
daily	18 (36.0%)		
≥10 times/day	0 (0.0%)		
**Tobacco use during pregnancy**			
**In the first trimester**	*N* = 123	*N* = 191	
yes	107 (87.0%)	191 (100.0%)	**<0.00001**
no	1 (0.8%)	0 (0.0%)	
unknown	15 (12.2%)	0 (0.0%)	
daily	53 (49.5%)	128 (67.0%)	**<0.00001**
≥10 cigarettes/day	26 (24.3%)	54 (28.3%)	0.16
**In the second trimester**	*N* = 76	*N* = 157	
yes	62 (81.6%)	144 (91.7%)	**0.02**
no	7 (9.2%)	9 (5.7%)	
unknown	7 (9.2%)	4 (2.6%)	
daily	28 (45.2%)	91 (63.2%)	**0.002**
≥10 cigarettes/day	10 (16.1%)	25 (17.4%)	0.98
**In the third trimester**	*N* = 73	*N* = 146	
yes	56 (76.7%)	125 (85.6%)	0.10
no	10 (13.7%)	11 (7.5%)	
unknown	7 (9.6%)	10 (6.9%)	
daily	30 (53.6%)	80 (64.0%)	0.06
≥10 cigarettes/day	12 (21.4%)	21 (16.8%)	0.69

Data reported as *N* (%) with *p*-value (exact Chi-square). Daily exposure: at least one consumption by day. Abbreviations: C: cannabis, T: tobacco. Use in the second and third trimester refers to pregnancies continued in the second and third trimester, respectively. *p*-value in bold: significant effect.

## Data Availability

The data are not publicly available due to privacy or ethical restrictions.

## Supplementary Materials

The following supporting information can be downloaded at: https://www.mdpi.com/article/10.3390/ijerph20176686/s1, Table S1: Logistic regression models for voluntary interruption of pregnancy by groups. Table S2: Logistic regression models for at least one event during pregnancy by groups. Table S3: Logistic regression models for composite adverse pregnancy outcome by groups. Table S4: Logistic regression models for gestational diabetes by groups. Table S5: Logistic regression models for at least one neonatal event by groups. Table S6: Logistic regression models for prematurity by groups. Table S7: Logistic regression models for small for gestational age by groups. Table S8: Logistic regression models for small for congenital malformations by groups. Table S9: Description of congenital malformations by groups. Table S10: Description of adverse events (AEs) in the C ± T group.

## References

[1] Spilka S., Richard J.B., Le Nezet O., Janssen E., Brissot A., Philipon A., Shah J., Chyderiotis S., Andler R., Cogordan C. (2018). Les Niveaux d’usages des Drogues Illicites en France en 2017—Tendances 128. Observatoire Français des Drogues et Toxicomanies et Santé Publique France. https://www.ofdt.fr/publications/collections/periodiques/lettre-tendances/les-niveaux-dusages-des-drogues-illicites-en-france-en-2017-tendances-128-novembre-2018/.

[2] INSERM—DREES—Santé Publique France (2022). Enquête Nationale Périnatale. Rapport 2021. Les Naissances, Le Suivi à Deux Mois et les établissements. Situation et évolution Depuis 2016. https://www.santepubliquefrance.fr/les-actualites/2022/sante-publique-france-partenaire-de-la-6e-edition-de-l-enquete-nationale-perinatale.

[3] Blayac L., Ponte C., Lavaud M., Micallef J., Lapeyre-Mestre M. (2022). Increase of cannabis and cocaine use by pregnant women in France from 2005 to 2018: Insights of the annual cross sectional OPPIDUM survey. Therapie.

[4] Bouquet E., Eiden C., Fauconneau B., Pion C., Pain S., Perault-Pochat M.C., French Addictovigilance Network (FAN) (2022). Adverse events of recreational cannabis use during pregnancy reported to the French Addictovigilance Network between 2011 and 2020. Sci. Rep..

[5] Sa S., Fonseca B.M., Martin C.R., Patel V.B., Preedy V.R. (2023). Cannabis consumption in reproductive function and teratogenicity. Cannabis Use, Neurobiology, Psychology and Treatment.

[6] Maia J., Almada M., Midão L., Fonseca B.M., Braga J., Gonçalves D., Teixeira N., Correia-da-Silva G. (2020). The Cannabinoid Delta-tetrahydrocannabinol Disrupts Estrogen Signaling in Human Placenta. Toxicol. Sci..

[7] Ayonrinde O.T., Ayonrinde O.A., Van Rooyen D.V., Tait R., Dunn M., Mehta S., White S., Ayonrinde O.K. (2021). Association between gestational cannabis exposure and maternal, perinatal, placental, and childhood outcomes. J. Dev. Orig. Health Dis..

[8] Cherki S. (2022). Le Point SINTES N°8. Observatoire Français des Drogues et Toxicomanies. https://www.ofdt.fr/BDD/sintes/LePointSINTES08.pdf.

[9] Dujourdy L., Besacier F. (2017). A study of cannabis potency in France over a 25 years period (1992–2016). Forensic Sci. Int..

[10] Madec J., Bouzillé G., Riou C., Van Hille P., Merour C., Artigny M.L., Delamarre D., Raimbert V., Lemordant P., Cuggia M. (2019). eHOP clinical data warehouse: From a prototype to the creation of an inter-regional clinical data centers network. Stud. Health Technol. Inform..

[11] Douchet M.A. (2023). Tabagisme et arrêt du Tabac en 2022. Observatoire Français des Drogues et des Tendances Addictives. http://www.ofdt.fr/ofdt/fr/tt_23bil.pdf.

[12] Saurel-Cubizolles M.J., Prunet C., Blondel B. (2014). Cannabis use during pregnancy in France in 2010. BJOG.

[13] Rodriguez C.E., Sheeder J., Allshouse A.A., Scott S., Wymore E., Hopfer A., Hermesch A., Metz T.D. (2019). Marijuana use in young mothers and adverse outcomes: A retrospective cohort study. BJOG.

[14] Heude B., Thiebaugeorges O., Goua V., Forhan A., Kaminski M., Foliguet B., Scheitzer M., Magnin G., Charles M.A., EDEN Mother-Child Cohort Study Group (2012). Pre-pregnancy body mass index and weight gain during pregnancy: Relations with gestational diabetes and hypertension, and birth outcomes. Matern. Child Health J..

[15] Block S.R., Watkins S.M., Salemi J.L., Rutkowski R., Tanner J.P., Correia J.A., Kirby R.S. (2013). Maternal pre-pregnancy body mass index and risk of selected birth defects: Evidence of a dose-response relationship. Paediatr. Perinat. Epidemiol..

[16] Corsi D.J. (2020). Epidemiological challenges to measuring prenatal cannabis use and its potential harms. BJOG.

[17] Corsi D.J., Hsu H., Weiss D., Fell D.B., Walker M. (2019). Trends and correlates of cannabis use in pregnancy: A population based study in Ontario, Canada from 2012 to 2017. Can. J. Public Health.

[18] Michalski C.A., Hung R.J., Seeto R.A., Dennis C.L., Brooks J.D., Henderson J., Levitan R., Lye S.J., Mathews S.G., Knight J.A. (2020). Association between maternal cannabis use and birth outcomes: An observational study. BMC Pregnancy Childbirth.

[19] Embersin-Kyprianou C., Yermachenko A., Massari V., El-Khoury-Lesueur F., Melchior M. (2020). Unexpected pregnancy, experience of sexual violence and contraception among women who use cannabis or other illegal substance in the Great Paris Region: Data from the 2016 Health Barometer. Rev. Epidemiol. Sante Publique.

[20] Singh S., Filion K.B., Abenhaim H.A., Eisenberg M.J. (2020). Prevalence and outcomes of prenatal recreational cannabis use in high-income countries: A scoping review. BJOG.

[21] Bandoli G., Jelliffe-Pawlowski L., Schumacher B., Baer R.J., Felder J.N., Fuchs J.D., Oltman S.P., Steurer M.A., Marienfeld C. (2021). Cannabis-related diagnosis in pregnancy and adverse maternal and infant outcomes. Drug Alcohol Depend..

[22] Luke S., Hobbs A.J., Smith M., Riddell C., Murphy P., Agborsangaya C., Cantin C., Fahey J., Der K., Pederson A. (2022). Cannabis use in pregnancy and maternal and infant outcomes: A Canadian cross-jurisdictional population-based cohort study. PLoS ONE.

[23] Prewitt K.C., Hayer S., Garg B., Benson A., Hedges M., Caughey A., Lo J.O. (2023). Impact of Prenatal Cannabis Use Disorder on Perinatal Outcomes. J. Addict. Med..

[24] El Marroun H., Tiemeier H., Steegers E.A.P., Jaddoe V.W.V., Hofman A., Verhulst F.C., van den Brink W., Huizink A.C. (2009). Intrauterine cannabis exposure affects fetal growth trajectories: The Generation R Study. J. Am. Acad. Child. Adolesc. Psychiatry.

[25] Metz T.D., Borgelt L.M. (2018). Marijuana use in pregnancy and while breastfeeding. Obstet. Gynecol..

[26] Risidzé M., Gargaros A., Fébrissy C., Dubucs C., Weyl A., Ousselin J., Aziza J., Arnal J.F., Lenfant F. (2023). Estrogen actions in placental vascular morphogenesis and spiral artery remodeling: A comparative view between Humans and Mice. Cells.

[27] Gong H., Chen Y., Xu J., Xie X., Yu D., Yang B., Kuang H. (2017). The regulation of ovary and conceptus on the uterine natural killer cells during early pregnancy. Reprod. Biol. Endocrinol..

[28] Wuu J., Hellerstein S., Lipworth L., Wide L., Xu B., Yu G.P., Kuper H., Lagiou P., Hankinson S.E., Ekbom A. (2002). Correlates of pregnancy oestrogen, progesterone and sex-hormon-binding globulin in the USA and China. Eur. J. Cancer Prev..

[29] Bemanian M., Vold J.H., Chowdhury R., Aas C.F., Gjestad R., Johansson K.A., Fadnes L.T. (2022). Folate Status as a Nutritional Indicator among People with Substance Use Disorder; A Prospective Cohort Study in Norway. Int. J. Environ. Res. Public Health.

[30] Williams P.J., Bulmer J.N., Innes B.A., Broughton Pipkin F. (2011). Possible roles for folic acid in the regulation of trophoblast invasion and placental development in normal early human pregnancy. Biol. Reprod..

[31] Stone R. (2015). Pregnant women and substance use: Fear, stigma, and barriers to care. Health Justice.

[32] Bouquet E., Pain S., Eiden C., Jouanjus E., Richard N., Fauconneau B., Pérault-Pochat M.C., French Addictovigilance Nework (2021). Adverse events of recreational cannabis use reported to the French addictovigilance network (2012–2017). Br. J. Clin. Pharmacol..

