# Supplementary material for: Maternal, Fetal and Neonatal Outcomes Related to Recreational Cannabis Use during Pregnancy: Analysis of a Real-World Clinical Data Warehouse between 2010 and 2019
Source: Int J Environ Res Public Health. 2023 Aug 30;20(17):6686. doi: 10.3390/ijerph20176686 (PMC10487904; doi:10.3390/ijerph20176686)
Supplement: Supplementary file 1 [file ijerph-20-06686-s001.zip › ijerph-2507474-supplementary.pdf]

Table S1: Logistic regression models for voluntary interruption of pregnancy by groups

Voluntary interruption pregnancy (N=121)	Unadjusted					Adjusted				
	**N**	**%**	**OR**	**IC 95% OR**	**p-value***	**N**	**%**	**ORa**	**IC 95% OR**	**p-value***
**C±T group**	40	33.1	**2.80**	**1.62-4.90**	0.00004	39	34.2	**3.90**	**1.48-10.84**	**0.02**
**T group**	28	23.1	ref			27	23.7	ref		
**CTRL group**	53	43.8	1.02	0.63-1.69		48	42.1	**2.72**	**1.07-7.20**	
**C±T group**	40	33.1	**2.75**	**1.70-4.42**	0.00004	39	34.2	1.44	0.61-3.40	
**T group**	28	23.1	0.98	0.59-1.59		27	23.7	**0.37**	**0.14-0.93**	
**CTRL group**	53	43.8	ref			48	42.1	ref		
Maternal age at birth										
< 25 years						63	55.3	ref		0.002
≥ 25 years						51	44.7	1.28	0.50-3.41	
Gynaeco-obtetrical history										
yes						84	73.7	0.40	0.23-0.68	0.001
no						30	26.3	ref		
Social coverage										
No health insurance						101	88.6	ref		<0.00001
Health insurance						8	7.0	**0.05**	**0.02-0.10**	
CMUc/AME						5	4.4	**0.10**	**0.03-0.24**	
**Maternal age at birth < 25 years**										
**C±T group**	26	39.7	1.03	0.52-2.04	**0.01**	25	39.7	**3.86**	**1.46-10.87**	**0.02**
**T group**	11	15.9	ref			10	15.9	ref		
**CTRL group**	32	44.4	**0.33**	**0.14-0.73**		28	44.4	**2.57**	**1.02-6.84**	
**C±T group**	26	39.7	**3.51**	**1.64-7.33**	**0.01**	23	39.7	1.50	0.64-3.59	0.02
**T group**	11	15.9	1.75	0.88-3.44		10	15.9	**0.39**	**0.15-0.98**	
**CTRL group**	32	44.4	ref			28	44.4	ref		
Gynaeco-obtetrical history										
yes						12	19.0	**0.28**	**0.12-0.64**	0.003
no						51	81.0	ref		
Social coverage										
No health insurance						4	6.3	**17.11**	**5.59-75.22**	<0.00001
Health insurance						56	4.8	ref		
CMUc/AME						3	88.9	**2.46**	**0.48-13.99**	
**Maternal age at birth ≥ 25 years**										
**C±T group**	14	26.9	2.00	0.90-4.37	**0.004**	14	27.5	1.52	0.60-3.82	**0.03**
**T group**	17	32.7	ref			17	33.3	ref		
**CTRL group**	21	40.4	0.57	0.29-1.13		20	39.2	0.47	0.21-1.04	
**C±T group**	14	26.9	**3.51**	**1.64-7.33**	**0.004**	14	27.5	**3.22**	**1.30-8.02**	**0.03**
**T group**	17	32.7	1.75	0.88-3.44		17	33.3	2.12	0.96-4.69	
**CTRL group**	21	40.4	ref			20	39.2	ref		
Gynaeco-obtetrical history										
yes						18	35.3	0.52	0.24-1.06	0.08
no						33	64.7	ref		
Social coverage										
No health insurance						1	2.0	**23.74**	**9.84-70.95**	<0.00001
Health insurance						5	9.8	ref		
CMUc/AME						45	88.2	1.13	0.06-7.34	

Abbreviations: CI: confidence interval; OR: odds ratio; ORa: adjusted odds ratio; VIP: voluntary interruption of pregnancy; CMUc: Supplementary Health-Care Coverage; AME: state medical assistance; C: cannabis; T: tobacco; CTRL: control; * Wald test

Table S2: Logistic regression models for at least one adverse event during pregnancy by groups

At least one event during pregnancy N=308	Unadjusted					Unadjusted (sub-population with complete BMI data)					Adjusted				
	N	%	OR	IC 95% OR	p-value*	N	%	OR	IC 95% OR	p-value*	N	%	OR_a	IC 95% OR	p-value*
**C±T group**	70	22.7	0.94	0.59-1.50	**<0.00001**	43	18.7	1.41	0.74-2.73	**0.008**	43	18.7	1.70	0.88-3.37	**0.002**
**T group**	111	36.0	ref			90	39.1	ref			90	39.1	ref		
**CTRL group**	127	41.2	**0.38**	**0.26-0.55**		97	42.2	**0.60**	**0.38-0.93**		97	42.2	**0.58**	**0.36-0.92**	
**C±T group**	70	22.7	**2.47**	**1.62-3.80**	**<0.00001**	43	18.7	**2.34**	**1.28-4.43**	**0.008**	43	18.7	**2.93**	**1.54-5.79**	**0.002**
**T group**	111	36.0	**2.63**	**1.83-3.81**		90	39.1	**1.66**	**1.07-2.59**		90	39.1	**1.73**	**1.08-2.78**	
**CTRL group**	127	41.2	ref			97	42.2	ref			97	42.2	ref		
Maternal age at birth (years)											230	100.0	**1.05**	**1.01-1.10**	**0.01**
Pre-pregnancy BMI (kg/m2)											230	100.0	**1.05**	**1.01-1.09**	**0.01**
Nulliparity															
yes											117	50.9	**1.80**	**1.15-2.84**	**0.01**
no											113	49.1	ref		

Single pregnancies; Abbreviations: CI: confidence interval; OR: odds ratio; OR_a: adjusted odds ratio; BMI: body mass index; C: cannabis; T: tobacco; CTRL: control;* Wald test

Table S3: Logistic regression models for composite adverse pregnancy outcome by groups

<u>Composite adverse pregnancy outcome</u> N=186	Univariate					Adjusted				
	N	%	OR	IC 95% OR	p-value*	N	%	OR_a	IC 95% OR_a	p-value*
**C±T group**	62	33.33	1.12	0.62-2.04	<0.00001	31	18.5	0.86	0.03-22.17	**<0.00001**
**T group**	84	45.16	ref			80	47.6	ref		
**CTRL group**	40	21.51	**0.20**	**0.13-0.31**		57	33.9	3.81	0.33-44.10	
**C±T group**	62	33.33	**5.53**	**3.15-9.87**	<0.00001	31	18.5	0.23	0.01-5.22	**<0.00001**
**T group**	84	45.16	**4.95**	**3.22-7.68**		80	47.6	0.26	0.07-2.88	
**CTRL group**	40	21.51	ref			57	33.9	ref		
Maternal age at birth (years)						168	100.0	**1.07**	**1.01-1.15**	0.29
Nulliparous										
yes						93	55.4	**1.72**	**1.08-2.74**	**0.02**
no						75	44.6	ref		
Social coverage										
No health insurance						19	11.3	**1.69**	**1.04-2.74**	0.06
Health insurance						89	53.0	ref		
CMUc/AME						60	35.7	0.90	0.44-1.77	
**≤ 28 years (N=89)**										
**C±T group**	18	21.4	1.01	0.48-2.17	**0.001**	18	22.2	1.01	0.43-2.40	**0.002**
**T group**	38	45.2	ref			37	45.7	ref		
**CTRL group**	28	33.3	0.35	0.19-0.66		26	32.1	0.34	0.18-0.66	
**C±T group**	18	21.4	**2.87**	**1.36-6.10**	**0.001**	18	22.2	**2.94**	**1.27-6.94**	**0.002**
**T group**	38	45.2	**2.82**	**1.51-5.34**		37	45.7	**2.90**	**1.51-5.70**	
**CTRL group**	28	33.3	ref			26	32.1	ref		
Maternal age at birth (years)						81	100.0	1.03	0.92-1.15	0.61
Nulliparous										
yes						55	67.9	1.39	0.71-2.75	0.34
no						26	32.1	ref		
Social coverage										
No health insurance						37	45.7	1.93	0.97-3.87	0.06
Health insurance						35	43.2	ref		
CMUc/AME						9	11.1	0.74	0.28-1.83	
**> 28 years (N=96)**										
**C±T group**	18	18.8	1.67	0.61-5.08	**<0.00001**	13	14.9	1.14	0.38-3.68	**<0.00001**
**T group**	45	46.9	ref			43	49.4	ref		
**CTRL group**	33	34.4	**0.12**	**0.06-0.22**		31	35.6	**0.13**	**0.07-0.25**	
**C±T group**	18	18.8	**13.6**	**5.28-40.07**	**<0.00001**	13	14.9	**8.64**	**3.04-27.11**	**<0.00001**
**T group**	45	46.9	**8.18**	**4.46-15.39**		43	49.4	**7.56**	**4.03-14.56**	
**CTRL group**	33	34.4	ref			31	35.6	ref		
Maternal age at birth (years)						87	100.0	1.00	0.92-1.10	0.91
Nulliparous										
yes						38	43.7	**2.20**	**1.16-4.19**	**0.02**
no						49	56.3	ref		
Social coverage										
No health insurance						10	11.5	1.39	0.67-2.82	0.60
Health insurance						54	62.1	ref		
CMUc/AME						23	26.4	1.42	0.48-4.09	

Single pregnancies; Abbreviations: CI: confidence interval; OR: odds ratio; OR_a: adjusted odds ratio; C: cannabis; T: tobacco; CTRL: control; CMUc: Complementary Universal Health Coverage; AME: state medical assistance; * Wald test

Table S4: Logistic regression models for gestational diabetes by groups

Gestational diabetes N=63	Unadjusted					Unadjusted (sub-population with complete BMI data)					Adjusted				
	N	%	OR	IC 95% OR	p-value*	N	%	OR	IC 95% OR	p-value*	N	%	OR_a	IC 95% OR_a	p-value*
**C±T group**	11	17.5	1.19	0.52-2.62	0.73	10	18.5	1.79	0.73-4.24	0.41	10	18.5	2.51	0.95-6.51	0.11
**T group**	19	30.2	ref			16	29.6	ref			16	29.6	ref		
**CTRL group**	33	52.4	0.89	0.49-1.65		28	51.9	1.15	0.60-2.27		28	51.9	1.02	0.50-2.12	
**C±T group**	11	17.5	1.33	0.62-2.72	0.73	10	18.5	1.56	0.67-3.40	0.41	10	18.5	1.91	0.82-4.28	
**T group**	19	30.2	1.12	0.60-2.03		16	29.6	0.87	0.67-3.40		16	29.6	1.16	0.60-2.19	
**CTRL group**	33	52.4	ref			28	51.9	ref			28	51.9	ref		
Maternal age at birth (years)											54	100.0	**1.12**	**1.05-1.19**	
Pre-pregnancy BMI (kg/m2)											54	100.0	**1.08**	**1.04-1.13**	
Gynaeco-obtetrical history															
yes											34	63.0	**2.03**	**1.09-3.88**	
no											20	37.0	ref		

Single pregnancies; Abbreviations: CI: confidence interval; OR: odds ratio; OR_a: adjusted odds ratio; BMI: body mass index; C: cannabis; T: tobacco; CTRL: control;* Wald test

Table S5: Logistic regression models for at least one neonatal event by groups

At least one neonatal event, N=189	Unadjusted					Unadjusted (sub-population with complete pre-pregnancy BMI data)					Adjusted				
	N	%	OR	IC 95% OR	p-value	N	%	OR	IC 95% OR	p-value*	N	%	OR_a	IC 95% OR_a	p-value*
**C±T group**	41	21.7	1.49	0.80-2.82	**<0.00001**	31	21.8	**2.38**	**1.07-5.76**	**<0.00001**	31	21.8	**2.29**	**1.02-5.58**	**<0.00001**
**T group**	76	40.2	ref			65	45.8	ref			65	45.8	ref		
**CTRL group**	72	38.1	**0.27**	**0.17-0.41**		46	32.4	**0.24**	**0.14-0.39**		46	32.4	0.24	0.14-14.74	
**C±T group**	41	21.7	**5.56**	**3.11-10.19**	**<0.00001**	31	21.8	**10.11**	**4.65-24.04**	**<0.00001**	31	21.8	**9.39**	**4.22-22.81**	**<0.00001**
**T group**	76	40.2	**3.73**	**2.42-5.79**		65	45.8	**4.24**	**2.57-7.08**		65	45.8	**4.10**	**2.43-7.00**	
**CTRL group**	72	38.1	ref			46	32.4	ref			46	32.4	ref		
Maternal age at birth (years)											142	100.0	0.99	0.94-1.03	0.55
Newborn sex															
girl											73	51.4	ref		0.29
boy											69	48.6	0.77	0.47-1.24	
Pre-pregnancy BMI (kg/m2)											142	100.0	0.99	0.95-1.03	0.60
Gynaeco-obtetrical history															
yes											67	47.2	1.28	0.78-2.11	0.32
no											75	52.8	ref		

Single pregnancies; Abbreviations: CI: confidence interval; OR: odds ratio; OR_a: adjusted odds ratio; BMI: body mass index; C: cannabis; T: tobacco; CTRL: control;* Wald test

Table S6: Logistic regression models for prematurity by groups

Prematurity N=91	Unadjusted					Adjusted				
	N	%	OR	IC 95% OR	p-value*	N	%	OR_a	IC 95% OR_a	p-value*
**C±T group**	28	30.8	1.71	0.91-3.21	**<0.00001**	25	27.5	1.65	0.70-3.84	0.85
**T group**	38	41.8	ref			38	41.8	ref		
**CTRL group**	25	27.5	**0.29**	**0.17-0.50**		28	30.8	0.53	0.24-1.16	
**C±T group**	28	30.8	**5.87**	**3.10-11.17**		25	27.5	**3.11**	**1.32-7.41**	
**T group**	38	41.8	**3.43**	**2.00-5.93**		38	41.8	1.89	0.86-4.20	
**CTRL group**	25	27.5	ref			28	30.8	ref		
Maternal age at birth										
≤ 28 years						45	49.5	ref		0.07
> 28 years						46	50.5	0.51	0.23-1.12	
**≤ 28 years (N=45)**										
**C±T group**	14	31.1	1.59	0.67-3.72	**0.03**	14	31.1	1.59	0.67-3.72	**0.05**
**T group**	17	37.8	ref			17	37.8	ref		
**CTRL group**	14	31.1	0.54	0.24-1.19		14	31.1	0.54	0.24-1.19	
**C±T group**	14	31.1	**3.11**	**1.32-7.41**	**0.03**	14	31.1	**2.93**	**1.23-7.03**	**0.05**
**T group**	17	37.8	1.89	0.86-4.20		17	37.8	1.84	0.84-4.10	
**CTRL group**	14	31.1	ref			14	31.1	ref		
Maternal age at birth (years)						45	100.0	0.94	0.84-1.06	0.32
**> 28 years (N=46)**										
**C±T group**	11	23.9	2.14	0.80-5.80	**<0.00001**	11	23.9	2.10	0.78-5.70	**<0.00001**
**T group**	21	45.7	ref			21	45.7	ref		
**CTRL group**	14	30.4	0.18	0.08-0.38		14	30.4	0.18	0.08-0.38	
**C±T group**	11	23.9	**11.7**	**4.36-32.65**	**<0.00001**	11	23.9	**11.47**	**4.23-31.87**	**<0.00001**
**T group**	21	45.7	**5.50**	**2.62-11.90**		21	45.7	**5.47**	**2.60-11.84**	
**CTRL group**	14	30.4	ref			14	30.4	ref		
Maternal age at birth (years)						46	100.	0.96	0.86-1.06	**0.40**

Single pregnancies; Abbreviations: CI: confidence interval; OR: odds ratio; OR_a: adjusted odds ratio; C: cannabis; T: tobacco; CTRL: control;* Wald test

Table S7: Logistic regression models for small for gestational age by groups

Small for gestational age N=89	Unadjusted					Unadjusted (sub-population with complete pre-pregnancy BMI data)					Adjusted				
	N	%	OR	IC 95% OR	p-value*	N	%	OR	IC 95% OR	p-value*	N	%	OR_a	IC 95% OR	p-value*
**C±T group**	22	24.7	1.03	0.55-1.91	**<0.00001**	16	23.5	1.28	0.61-2.65	**<0.00001**	16	23.5	1.14	0.53-2.42	**<0.00001**
**T group**	47	52.8	ref			38	55.9	ref			38	55.9	ref		
**CTRL group**	20	22.5	**0.15**	**0.08-0.27**		14	20.6	**0.17**	**0.08-0.32**		14	20.6	**0.16**	**0.08-0.32**	
**C±T group**	22	24.7	**6.80**	**3.43-13.62**	**<0.00001**	16	23.5	**7.56**	**3.32-17.55**	**<0.00001**	16	23.5	**7.15**	**3.04-17.16**	**<0.00001**
**T group**	47	52.8	**6.58**	**3.76-11.89**		38	55.9	**5.91**	**3.09-11.87**		38	55.9	**6.26**	**3.17-13.03**	
**CTRL group**	20	22.5	ref			14	20.6	ref			14	20.6	ref		
Maternal age at birth (years)											68	100.0	1.03	0.97-1.09	0.31
Pre-pregnancy BMI (kg/m2)											68	100.0	0.96	0.91-1.01	0.13
Newborn sex															
girl											38	55.9	ref		0.21
boy											30	44.1	0.69	0.39-1.23	
Nulliparity															
yes											44	64.7	**2.10**	**1.14-3.93**	**0.02**
no											24	35.3	ref		

Single pregnancies; Abbreviations: CI: confidence interval; OR: odds ratio; OR_a: adjusted odds ratio; BMI: body mass index; C: cannabis; T: tobacco; CTRL: control; * Wald test

Table S8: Logistic regression models for congenital malformations by groups

Congenital malformations, N=47	Unadjusted					Unadjusted (sub-population with complete pre-pregnancy BMI data)					Adjusted				
	N	%	OR	IC 95% OR	p-value*	N	%	OR	IC 95% OR	p-value*	N	%	OR_a	IC 95% OR_a	p-value*
**C±T group**	10	21.3	1.74	0.71-4.20	0.25	8	20.5	1.94	0.71-5.09	0.28	8	20.5	2.01	0.73-5.32	0.28
**T group**	13	27.7	ref			12	30.8	ref			12	30.8	ref		
**CTRL group**	24	51.1	0.89	0.45-1.86		19	48.7	0.96	0.45-2.11		19	48.7	0.98	0.45-2.19	
**C±T group**	10	21.3	1.95	0.85-4.20	0.25	8	20.5	2.02	0.78-4.85	0.28	8	20.5	2.05	0.77-5.09	0.28
**T group**	13	27.7	1.12	0.54-2.23		12	30.8	1.04	0.47-2.21		12	30.8	1.02	0.46-2.22	
**CTRL group**	24	51.1	réf			19	48.7	ref			19	48.7	ref		
Maternal age at birth (years)											39	100.0	1.00	0.94-1.06	0.98
Pre-pregnancy BMI (kg/m2)											39	100.0	1.01	0.96-1.07	0.59

Single pregnancies; Abbreviations: CI: confidence interval; OR: odds ratio; OR_a: adjusted odds ratio; BMI: body mass index; C: cannabis; T: tobacco; CTRL: control * Wald test

Table S9. Description of congenital malformations by groups

Highlighted in grey: medical termination of pregnancy

N° of cases	C±T group (n=10, 21.3%)	T group (n=13, 27.7%)	CTRL group (n=24, 51.1%)
1	- **cardiac**: atrial septal defect	- **cardiac** : large ductus arteriosus and atrial septal defect	- **cardiac** : ventricular septal defect
2	- **cardiac** : slightly thickened interventricular septum and atrial septal defect	- **cardiac :** atrial septal aneuvrysm with persistant foramen ovale	- **cardiac** : atrial septal defect
3	- **cardiac**: atrial septal defect<br>- **urinary** : hypotrophy of the right kidney	- **cardiac** : asymetry of the heart chambers, small ductus arteriosus	**- cardiac** : 2 interventricular septal defects and one atrial septal defect
4	- **cardiac :** hypoplasia of the left ventricles<br>- peri-thoracic oedema<br>**- urinary**: méga-bladder	- **cardiac** : atrial septal defect	- **cardiac** : dilatation of the right atrium<br>- **facial** : hygroma coli, facial dysmorphia<br>- **genital** : hypospadias
5	- **urinary**: right uropathy	- **cardiac** : perimembranous interventricular communication	- **cardiac** : atrial septal defect<br>- **osteoarticular** : foot slope
6	- **osteoarticular**: short femoral length (4th percentile)	- **cardiac** : subrenal agenesis of the inferior vena cava	- **urinary** : bilateral pyelectasis
7	- **osteoarticular**: right clubfoot	- **urinary** : left pyelectasis	- **urinary** : bilateral pyelectasis
8	**- osteoarticular**: right clubfoot<br>(according to the geneticist, most likely etiology if folic acid deficiency)<br>- **nervous system**: microcephaly, bilateral ventriculomegaly, Arnold Chiari 2 abnormality, thoracolumbar spina bifida aperta	- **urinary** : left renal agenesis, compensatory right renal hypertrophy	- **urinary** : bilateral pyelectasy
9	**- digestive** : small bowel atresia	- **urinary :** polycystic kidneys with uretreral and bladder dilatation, posterior ureteral valves, Potter sequence<br>- **pulmonary :** hypotrophy of the lung	- **urinary** : bilateral pyelocaliceal and ureteral dilatation
10	- **digestive** : enlarged gallbladder<br>- **facial** : lip occlusion defect, short nasal bones	- **digestive** : oesophageal atresia	- **urinary** : uropathy
11		- **Walker-Warburg syndrome**	- **genitals**: cryptorchidism
12		- **chromosomal** : Turner syndrome	- **nervous system** : proximal spinal muscular atrophy
13		- **chromosomal : Di Georges** syndrome	- **nervous system** : hydrocephalus with ventriculomegaly and septal rupture, cerebellar hypoplasia in the sustentorial spaces
14			- **nervous system**: spina bifida
15			- **facial**: bilateral cleft lip and palate
16			- **digestive**: laparoschisis
17			**- osteoarticular** : micromelic and platispondylic dwarfism (FGFR3 gene mutation)
18			**- chromosomic :** Noonan syndrome
19			**- chromosomic** : trisomy 13
20			**- chromosomic** : trisomy 22
21			**- chromosomic** : persistent cervical hygroma, suspected ventricular septal defects
22			**- other** : Left-handedness (Gaucher disease)
23			**- other :** Beckwith-Wiedemann syndrome
24			**- other**: congenital nevus

Table S10. Description of adverse events (AEs) in the C±T group

**Maternal AEs** (N=73)	**Fetal AEs** (N=13)	**Neonatal AEs** (N=79)
**Threat of preterm delivery (n=11)**<br>Gestational diabetes (n=11)<br>**Cannabis dependence (n=8)**<br>**Oligoamnios/anamnios (n=7)**<br>**Placental abnormalities (n=7): retroplacental hematoma (n=3), placenta previa (n=2), subchorionic hematoma et thrombosis (n=2)**<br>**Premature rupture of membranes (n=6)**<br>**Hypertension/Eclampsia/Pre-eclampsia/HELLP syndrome (n=5)**<br>**Ectopic pregnancy (n=4)**<br>**Maternal cannabis withdrawal syndrome (n=2)**<br>**Medical termination of pregnancy (n=2)**<br>**Miscarriage (n=2)**<br>**Hemorrhagic stroke (n=1)**<br>**Reversible posterior encephalopathy syndrome (n=1)**<br>**Clastic crisis (n=1)**<br>**Agitation and depressive syndrome (n=1)**<br>**Sad mood (n=1)**<br>**Adrenal necrosis (n=1)**<br>**Abdominal pain (n=1)**<br>**Folic acid deficiency (n=1)**	**Fetal heart rhythm disorder (n=12)**<br>**In utero fetal death (n=1)**	Prematurity (n=28)<br>Small for gestational age (n=22)<br>Congenital malformations (n=10)<br>**Apgar < 7 at 1 minute (n=6)**<br>**Respiratory failure (n=4)**<br>**Stillbirth (n=3)**<br>**Hypoglycemia (n=3)**<br>**Bradycardia (n=2)**<br>**Behaviour problem (n=1)**

Single pregnancies. Abbreviations: AE: adverse event
